# Implementation of pre-exposure prophylaxis programme in Spain. Feasibility of four different delivery models

**DOI:** 10.1371/journal.pone.0246129

**Published:** 2021-02-08

**Authors:** Carlos Iniesta, Pep Coll, María Jesús Barberá, Miguel García Deltoro, Xabier Camino, Gabriela Fagúndez, Asunción Díaz, Rosa Polo, Spanish Working Group for PrEP

**Affiliations:** 1 Spanish HIV/AIDS Research Network, National Centre of Epidemiology, Health Institute Carlos III, Madrid, Spain; 2 National AIDS Programme, Ministry of Health, Madrid, Spain; 3 BCN Checkpoint, Barcelona, España; 4 STI Unit Vall d´Hebron-Drassanes, Infectious Diseases Department, University Hospital Vall d’Hebron, Barcelona, Spain; 5 Infectious Disease Service, Consortium General University Hospital of Valencia, Valencia, Spain; 6 Infectious Disease Service, University Hospital of Donostia, San Sebastian, Spain; 7 HIV and STI Surveillance Unit, National Centre of Epidemiology, Health Institute Carlos III, Madrid, Spain; Beth Israel Deaconess Medical Center/Harvard Medical School, UNITED STATES

## Abstract

**Background:**

Pre-exposure prophylaxis (PrEP) is an effective and cost-effective strategy for HIV prevention. Spain carried out an implementation study in order to assess the feasibility of implementing PrEP programmes within its heterogeneous health system.

**Methods:**

Observational longitudinal study conducted on four different types of health-care setting: a community centre (CC), a sexually transmitted infections clinic (STIC), a hospital-based HIV unit (HBHIVU) and a hospital-based STI unit (HBSTIU). We recruited gay, bisexual and other men who have sex with men (GBSM) and transgender women at risk of HIV infections, gave them PrEP and monitored clinical, behavioural PrEP-related and satisfaction information for 52 weeks. We collected perceptions on PrEP implementation feasibility from health-care professionals participating in the study.

**Results:**

A total of 321 participants were recruited, with 99.1% being GBMSM. Overall retention was 87.2% and it was highest at the CC (92.6%). Condom use decreased during the study period, while STIs did not increase consistently. The percentage of people who did not miss any doses of PrEP during the previous week remained at over 93%. No HIV seroconversions occurred. We observed overall decreases in GHB (32.5% to 21.8%), cocaine (27.5% to 21.4%), MDMA (25.7% to 14.3%), speed (11.4% to 5.7%) and mephedrone use (10.7% to 5.0%). The overall participant satisfaction with PrEP was 98.6%. Health-care professionals’ perceptions of PrEP feasibility were positive, except for the lack of personnel.

**Conclusions:**

PrEP implementation is feasible in four types of health-care settings. Local specificities have to be taken into consideration while implementing PrEP.

## Introduction

Although there has been a decline in the incidence of HIV in recent years in Spain, 3244 new HIV diagnoses were reported in 2018, 56.4% of which were gay, bisexual or other men who have sex with men (GBMSM) [1]. These numbers reflect the need for new preventive strategies such as Pre-Exposure Prophylaxis (PrEP), which has proved to be safe, effective and cost-effective [2–4].

Despite the strong evidence available, PrEP implementation has been unequal across European countries [5]. Political and structural factors may explain this heterogeneous situation [6], but the need to generate context-specific evidence has also led to many countries setting up demonstration studies prior to effective PrEP implementation [7–9].

In 2016, the Spanish Agency of Medicines and Medical Devices (AEMPS) approved the commercial combination of tenofovir and emtricitabine for PrEP indication as part of a comprehensive prevention programme [10]. The National AIDS Programme at the Ministry of Health (MOH) also published national guidelines recommending PrEP for most-at-risk populations, especially GBMSM and transgender women (TW) [11]. Finally, daily PrEP with tenofovir disoproxil/emtricitabine, free of charge, was included in the National Health System (NHS) in November 2019 for GBMSM and female sex workers over eighteen years old.

Different barriers have operated along the pathway towards PrEP implementation. First, Spanish territory is divided into autonomous regions (CCAA) with their own health systems, which means that decisions on the implementation of new strategies at a national level must be agreed with them. Furthermore, the HIV epidemic is heterogeneous across CCAA, with the majority of new diagnoses concentrated in Madrid and Catalonia and fewer cases in other regions. Lastly, antiretrovirals are regulated as drugs for hospital use, and therefore can only be delivered in hospital pharmacies. Because of this, different possible models for PrEP delivery have been described within the NHS [12].

The National AIDS Programme decided to conduct a study with the general objective of assessing the feasibility of implementing PrEP in the NHS. Specific objectives included: 1) to assess the feasibility of setting up a PrEP programme in four different settings within the NHS; 2) to describe participant’s characteristics, retention in the study, reasons for abandonment and factors related to abandonment and STI incidence; 3) to assess changes in renal function, PrEP adherence, STI diagnosis and behavioural variables after one year of PrEP use, overall and by study setting; and 4) to know participants’ level of satisfaction and professionals’ perceptions about PrEP implementation feasibility.

## Methods

### Overview

Post-authorization, observational, longitudinal study. The recruitment period ran from December 2017 to October 2018. The study was designed by the Spanish National AIDS Programme, with a pragmatic perspective, and it was approved by the Ethics Committee of the Hospital Clínico San Carlos in Madrid. The follow-up period was 52 weeks.

### Study sites

There were four study sites, each of which was a different type of health-care setting: 1) a community-based centre (CC) for testing HIV and other sexually transmitted infections (STI), targeted towards GBMSM and TW situated in Barcelona, Catalonia; 2) an STI clinic (STIC), which belongs to one of the largest hospitals in the city of Barcelona but has its own facilities separate from it; 3) a hospital-based outpatient HIV unit (HBHIVU) in the city of Valencia; and 4) an outpatient STI unit (HBSTIU) in San Sebastian, which belongs to the city’s hospital but is located in a primary care facility. Characteristics of the study sites are detailed in Table 1.

**Table 1:** Characteristics of the study sites.

Study site	Ownership	Service Location	Medical card required	STI screening procedures	Support measures for drug users	PrEP delivery	Costs for PrEP drugs and
CC	NGO	Community centre facility	No	Anal and throat swab, urine PCR and blood sample at every visit	Counselling	On site	Free of charge
STIC	Public Hospital	Non hospital-based STI Clinic	No	Anal and throat swab, urine PCR, and blood sample at every visit	Counselling, referral to specific resources	On site	Free of charge
HBHIVU	Public Hospital	HIV Outpatient Unit at Hospital	Yes	Urine PCR and blood sample at every visit. Throat and anal swab only on symptomatic participants	None	On site	Free of charge
HBSTIU	Public Hospital	STI Outpatient Unit at a primary care facility	Yes	Anal and throat swab, urine PCR, and blood sample.	Counselling	On site	Free of charge

CC: Community centre; STIC: STI clinic; HBHIVU: hospital-based HIV unit; HBSTIU: hospital-based STI unit (HBSTIU)

### Participants

We aimed to recruit 400 participants. The inclusion criteria were: being a GBMSM, being a TW, being between 18 and 65 years old, having a negative fourth-generation ELISA test for HIV within a week prior to recruitment and being at risk of acquiring an HIV infection. Participants were considered to be at risk of acquiring HIV if they met two or more of the following criteria, referring to the last 12 months: had more than 10 sexual partners, had condomless anal intercourse, use of recreational drugs, use of post-exposure prophylaxis, or received a diagnosis of bacterial STI. The exclusion criteria were: being in a window period or having symptoms suggestive of HIV acute infection, having altered renal function, previous bone disease or liver disease, and not being reliable for good adherence levels according to provider judgement.

### Procedures

Once eligible participants signed the informed consent form, health-care providers from study centres collected socio-demographic information and baseline clinical and behavioural information. Three weeks later, a new HIV test was performed and those with a negative result were retained in the study and initiated PrEP. Visits occurred then at weeks 4, 12, 24, 36, 48 and 52. Daily PrEP was provided for 3 months and counselling on HIV and STI prevention was given at every visit. Clinical, behavioural, PrEP-related and satisfaction information was collected at every visit (see S1 Appendix).

#### Clinical measures

HIV, syphilis, chlamydia and gonorrhoea test results were recorded. Renal function was monitored regularly through two parameters: serum creatinine and estimated glomerular filtration rate (eGFR), which was categorized into under 60 mL/min (altered) or 60 mL/min or over (normal).

#### Behavioural measures

Participants were asked about their frequency of condom use during sexual intercourse. Those who answered “often” or “always” were considered to use condom regularly. Also, they were asked about their regular use of alcohol, cannabis, GHB/GBL, poppers, MDMA, ketamine, mephedrone, speed, methamphetamine and any other drugs.

#### PrEP-related measures

After PrEP uptake, participants were asked about their adherence to PrEP using the Simplified Medication Adherence Questionnaire (SMAQ). We analysed the global SMAQ score and one specific item referring to the number of missed doses during the last week. The main reasons for discontinuing PrEP were categorized as follows: moving to a different city, low exposure to HIV, side effects, low adherence and other reasons (which included patients’ decisions other than those described or health problems not related to the study). PrEP retention was calculated based on the participants that had completed the 52-week follow-up period.

#### Satisfaction measures

Participants and professionals’ satisfaction with the programme was measured by asking about their degree of agreement (four-point Likert scales: strongly agree, agree, disagree, strongly disagree) with different statements. In this work we present the following items: 1) satisfaction with waiting times; 2) satisfaction with the information received about collecting PrEP pills; 3) satisfaction with the information received about what to do in the case of side effects; 4) general satisfaction with PrEP. We calculated the percentage of participants that showed the highest possible degree of agreement. Professionals from each study site were asked to indicate the extent to which they agreed with the following five statements regarding feasibility: 1) Participants’ willingness to participate in the study was as high as expected; 2) The proportion of participants meeting PrEP criteria was similar to that expected; 3) The centre’s facilities allowed privacy to be maintained during visits; 4) The relationship with the hospital has been appropriate; 5) The centre has enough personnel to conduct the study.

### Data analysis

Statistical analyses were conducted using Stata version 14.1. We performed descriptive analyses for all variables. We used a multivariate logistic regression to identify factors related to study abandonment for any cause, using participant characteristics at baseline and the study site as exposure variables. Incidence rate of STI was calculated with censuring after a positive test. For those who finished the study, we analysed changes in the measures between baseline and the end of the study of clinical, behavioural and PrEP-related measures using the Bowker symmetry test for qualitative variables and student’s t-student for quantitative variables. Graphs were created using Prism GraphPad version 8.4.2. Data are available from https://doi.org/10.6084/m9.figshare.13365902.v1.

## Results

### Characteristics of recruited participants

A total of 321 participants were recruited. The site that recruited the biggest number of participants was the CC (175). The median age was 36 and 99% were GBMSM. Overall, 59.2% of the participants were Spanish. The highest proportion of foreigners was found in the STIC (46.0%), while only 8.0% of participants were foreigners in the HBHIVU. Some 67.9% had a university education and 86.6% were employed. The centres with the lowest levels of university education and employment were the HBSTIU and the STIC. A proportion of 40.0% of participants presented four or five of the risk behaviours that were considered inclusion criteria, with a higher proportion in the STIC (Table 2).

**Table 2:** Socio-demographic characteristics of participants.

		Overall (n = 321)	CC (n = 175)	STIC (n = 100)	HBHIVU (n = 25)	HBSTIU (n = 21)	p-value
Age*		36 (31‒42)	37 (31‒42)	36 (30‒41.5)	34 (30‒40)	40 (31‒43)	0.285
Key population	GBMSM	318 (99.1)	174 (99.4)	99 (99.0)	25 (100.0)	20 (95.2)	0.282
	TW	3 (0.9)	1 (0.6)	1 (1.0)	0 (0.0)	1 (4.8)	
Origin	Spanish	190 (59.2)	95 (54.3)	54 (54.0)	23 (92.0)	18 (85.7)	< 0.001
	Foreigner	131 (40.8)	80 (45.7)	46 (46.0)	2 (8.0)	3 (14.3)	
Education	Primary	19 (5.9)	2 (1.1)	11 (11.0)	3 (12.0)	3 (14.3)	0.004
	High school	84 (26.2)	45 (25.7)	26 (26.0)	5 (20.0)	8 (38.1)	
	University	218 (67.9)	128 (73.1)	63 (63.0)	17 (68.0)	10 (47.6)	
Working situation	Employed	278 (86.6)	159 (90.9)	79 (79.0)	23 (92.0)	17 (81.0)	0.030
	Unemployed	43 (13.4)	16 (9.1)	21 (21.0)	2 (8.0)	4 (12.1)	
Number of inclusion criteria met	2‒3	190 (59.1)	105 (60.0)	47 (47.0)	18 (72.0)	20 (95.2)	< 0.001
	4‒5	131 (40.8)	70 (40.0)	53 (53.0)	7 (28.0)	1 (4.8)	

* Median (interquartile range) CC: Community centre; STIC: STI Clinic; HBHIVU: Hospital-based HIV Unit. HBSTIU: Hospital-based STI Unit.

### Study flow, abandonments and side effects

Five participants that were initially considered for recruitment were HIV positive and therefore excluded. 321 participants were recruited: three of them were lost to follow-up before starting PrEP, 318 started PrEP and 280 finished the 52-week follow-up period (see S1 Fig), which was completed in October 2019. The overall retention rate of the study was 82.3% (Table 2), with the highest percentage in the CC (92.6%) and the lowest in the HBSTIU (71.4%) (p = 0.005).

Of those who abandoned the study, the most frequent reason was being lost to follow-up (LTFU) (36.6%), followed by moving to a different city (12.2%) and low exposure to HIV (12.2%, mostly due to engaging in a couple relationship during the study). Only four participants discontinued PrEP because of side effects: one participant presented a headache, one presented a skin rash and two presented gastrointestinal disorders (Table 3). Participants who finished the study maintained levels of eGFR over 60 ml/min, and the average serum creatinine went from 0.91 mg/dL (standard deviation [SD]: 0.13) to 0.93 md/dL (0.14 [SD: 0.13]), (p = 0.001).

**Table 3:** Frequency and reasons for abandonment.

	Overall (n = 321)	CC (n = 175)	STIC (n = 100)	HBHVU (n = 25)	HBSTIU (n = 21)
Percentage of retention					
Retention	280 (87.2%)	162 (92.6)	81 (81.0)	22 (88.0)	15 (71.4)
Frequency distribution for reasons for abandonment*					
LTFU	15 (36.6)	3 (23.1)	8 (42.1)	0 (0.0)	4 (66.7)
Moving to a different city	12 (29.3)	6 (46.2)	3 (15.8)	2 (66.7)	1 (16.7)
Low exposure to HIV	5 (12.2)	1 (7.7)	3 (15.8)	0 (0.0)	1 (16.7)
Side effects	4 (9.8)	2 (15.4)	1 (5.3)	1 (33.3)	0 (0.0)
Low adherence	1 (2.4)	0 (0.0)	1 (5.3)	0 (0.0)	0 (0.0)
Other reasons	4 (9.8)	1 (7.7)	3 (15.8)	0 (0.0)	0 (0.0)
Total	41 (100)	13 (100)	19 (100)	3 (100)	6 (100)

* Percentage among those who abandoned. CC: Community centre; STIC: STI Clinic; HBHIVU: Hospital-based HIV Unit. HBSTIU: Hospital-based STI Unit.

LTFU = Lost to follow-up.

At baseline, age, origin, university education, number of inclusion criteria met and diagnosis of STI were not related with a higher frequency of abandonment. The only factor related to abandonment was the study site, adjusted by age, origin and education: compared with the CC, participants from the HBHIVU (p = 0.010) and from the HBSTIU (p = 0.010) were more likely to abandon the study for any reason (Table 4).

**Table 4:** Univariable and multivariable analysis of baseline factors related to abandonment.

		cOR	p-value	aOR	p-value
Age*	45–65	1		1	
	30–44	1.06 (0.41–2.75)	0.902	0.95 (0.35–2.53)	0.913
	18–29	2.61 (0.92–7.43)	0.072	2.23 (0.76–6.55)	0.144
Origin	Foreigner	1		1	
	Spanish	0.92 (0.47–1.8)	0.803	1.03 (0.5–2.14)	0.932
University education*	No	1		1	
	Yes	0.63 (0.32–1.22)	0.171	0.7 (0.35–1.42)	0.327
Drug use*	No	1			
	1–2 drugs	1.37 (0.64–2.93)	0.419		
	3 or more drugs	1 (0.43–2.34)	0.993		
Inclusion criteria met	2 or 3	1			
	4 or 5	1.16 (0.6–2.24)	0.666		
STIs diagnoses *	No	1			
	Yes	1.27 (0.55–2.92)	0.580		
Study site	CC	1		1	
	STIC	1.7 (0.45–6.44)	0.435	1.6 (0.4–6.4)	0.504
	HBHIVU	2.92 (1.37–6.21)	0.005	2.72 (1.27–5.85)	0.010
	HBSTIU	4.98 (1.66–15.01)	0.004	4.44 (1.42–13.92)	0.010

* At baseline. CC: Community centre; STIC: STI Clinic; HBHIVU: Hospital-based HIV Unit. HBSTIU: Hospital-based STI Unit.

### PrEP adherence and condom use

At week 4, 75.9% of participants were adherent to PrEP according to SMAQ criteria. This percentage increased to 85.9% at week 52 (p = 0.007). However, the number of people who did not miss any dose during the week prior to the study visit was 93.2% at week 4 and remained at 94.3% at week 52 (p = 0.350). The trend remained stable in all the study centres. HBSTIU had the lowest percentage of participants not missing any dose at week 52, and STIC presented the highest (97.5%), (p = 0.003) (Table 5).

**Table 5:** Missing dose frequency during last week.

		Week 4	Week 52	*p‒value*
Global	None	261 (93.2)	264 (94.3)	*p = 0*.*350*
	1‒2	18 (6.4)	12 (4.3)	
	3‒4	(0.0)	1 (0.4)	
	5‒7	1 (0.4)	3 (1.1)	
CC	None	151 (93.2)	153 (94.4)	*p = 0*.*480*
	1‒2	10 (6.2)	6 (3.7)	
	3‒4	(0.0)	1 (0.6)	
	5‒7	1 (0.6)	2 (1.2)	
STIC	None	76 (93.8)	79 (97.5)	*p = 0*.*257*
	1‒2	5 (6.2)	2 (2.5)	
	3‒4	(0.0)	(0.0)	
	5‒7	(0.0)	(0.0)	
HBHIVU	None	19 (86.4)	21 (95.5)	*p = 0*.*135*
	1‒2	3 (13.6)	(0.0)	
	3‒4	(0.0)	(0.0)	
	5‒7	(0.0)	1 (4.6)	
HBSTIU	None	15 (100.0)	11 (73.3)	*p = 0*.*257*
	1‒2	(0.0)	4 (26.7)	
	3‒4	(0.0)	(0.0)	
	5‒7	(0.0)	(0.0)	

CC: Community‒based centre, STIC: STI clinic; HBHIVU: Hospital‒based HIV unit; HBSTIU: Hospital‒based STI unit.

A total of 66.1% of participants reported using condoms regularly at baseline. This percentage decreased to 37.8% at week 52. At week 52, participants from the CC showed the lowest rate of regular condom use (25.6%), while HBSTIU’s participants showed the highest rates (86.7%) (p<0.001). The observed decrease was significant globally (p<0.001) and for the CC (p<0.001), STIC (p = 0.049) and HBHIVU (p = 0.007).

### HIV seroconversions and STI diagnoses

**Fig 1:**
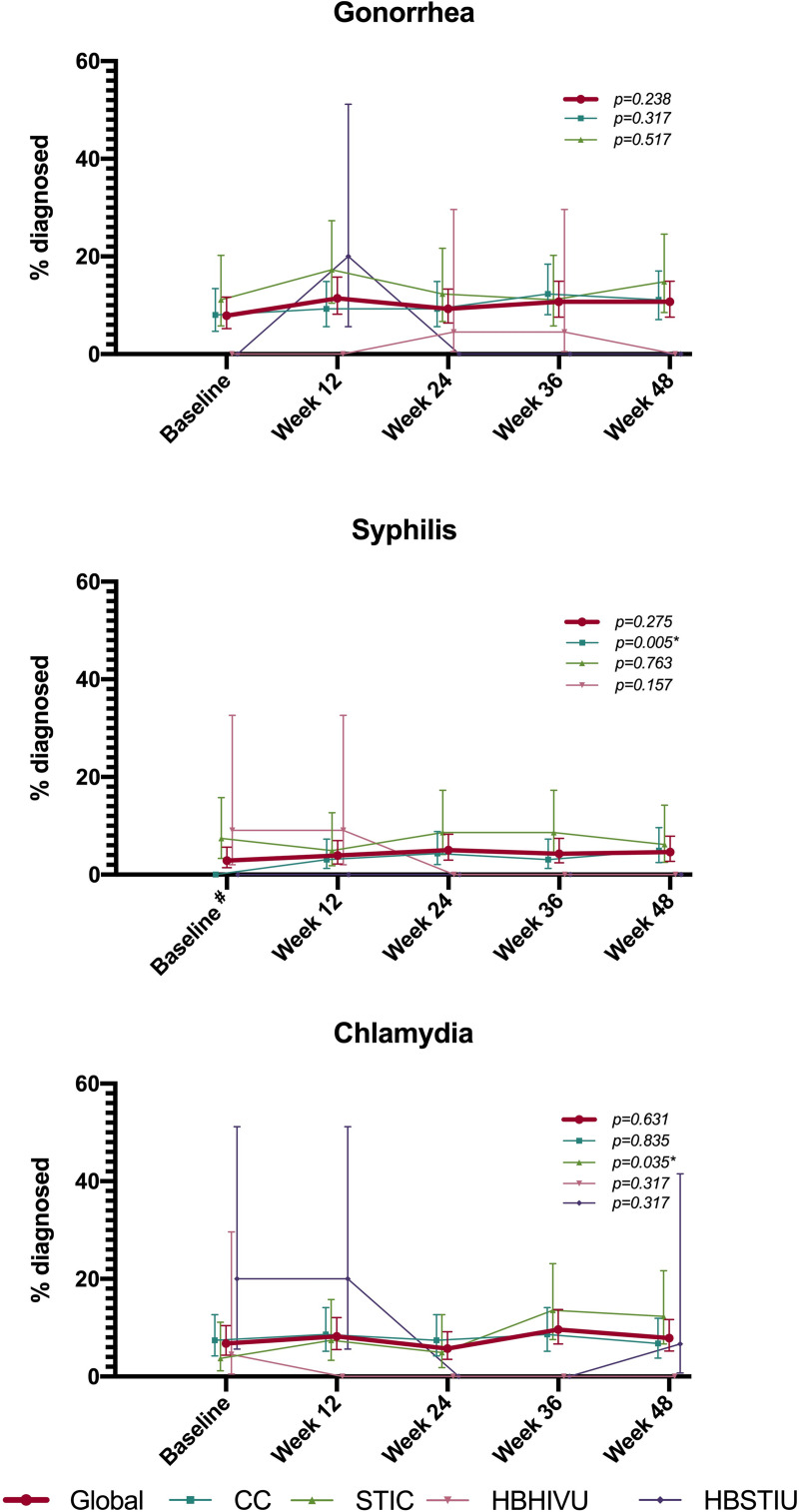
Percentage of participants diagnosed with STI. # p<0.005 for Chi-squared test for percentage difference between centers. p- values showed on the graph areas refer to the symmetry Bowker test to test the signifance of the increase between baseline and week 48 measures. * refers to statistically significant p-values.

No HIV seroconversions occurred during the study period. The baseline frequency of gonorrhoea diagnosis was 7.9% (95%CI: 5.2‒11.7), with no differences between study centres at any visit. The overall percentage of gonorrhoea diagnoses at week 48 was 10.7% (95%CI: 7.6‒15.0). This increase was not significant either globally or by centre. The frequency of syphilis diagnosis at baseline was 2.9% (95%CI: 1.4‒5.6), being highest at the HBHIVU (9.1%; 95%CI: 2.0‒32.7) and lowest at the CC and the HBSTIU (0 cases). This difference was significant (p = 0.003). At week 48, the frequency of syphilis diagnosis was 4.6% (95%CI: 2.7‒7.9). This increase was not significant for overall data, but it was for the CC, ranging from 0 cases at baseline to 4.9% (95%CI: 2.5‒9.6) at week 48. Overall, chlamydia infection was diagnosed in 6.8% of participants (95%CI: 4.3‒10.4) at baseline and in 7.9% (95%CI: 5.2‒11.7) at week 48, although this increase was not significant globally. However, we observed a significant increase among STIC participants, ranging from 3.7% (95%CI: 1.2‒11.1) of cases diagnosed with chlamydia at baseline to 12.4% (95%CI: 6.7‒21.7) at week 48 (Fig 1).

At week 48, the incidence of one or more STIs was 68.4 (95%CI:59.5–80.0) per 100 person-years. Among those who had an STI diagnosis, 54.8% (86) had only one STI, 28.7% (45) had two STIs, 12.7% (20) had three STIs and 3.8% (6) had four or more STIs.

The incidence of one or more episodes of gonorrhoea at week 48 was 38.24 (95%CI: 31.4–46.56) per 100 person-years. Among those who had at least one episode of gonorrhoea diagnosed, 78.8% (78) had only one episode, 18.2% (18) had two episodes, and 3.0% (3) had three episodes.

At week 48, the incidence of one or more episodes of syphilis was 15.99 (95%CI: 11.94–21.41) per 100 person-years. A total of 84.4% (38) of those who had a syphilis infection had one episode, and 15.6% (7) had two episodes of infection.

Chlamydia incidence at week 48 was 27.37 (21.8–34.38) per 100 person-years. Among those who had at least one episode of chlamydia diagnosed, 78.4% (58) had only one episode, 18.9% (14) had two episodes and 2.7% (2) had three episodes of chlamydia infection.

Globally, 4 participants had a positive serology for HCV at baseline. One participant negative at baseline seroconverted at week 12, which equals to an incidence of 6.57(0.93–46.66).

### Drug use

**Fig 2:**
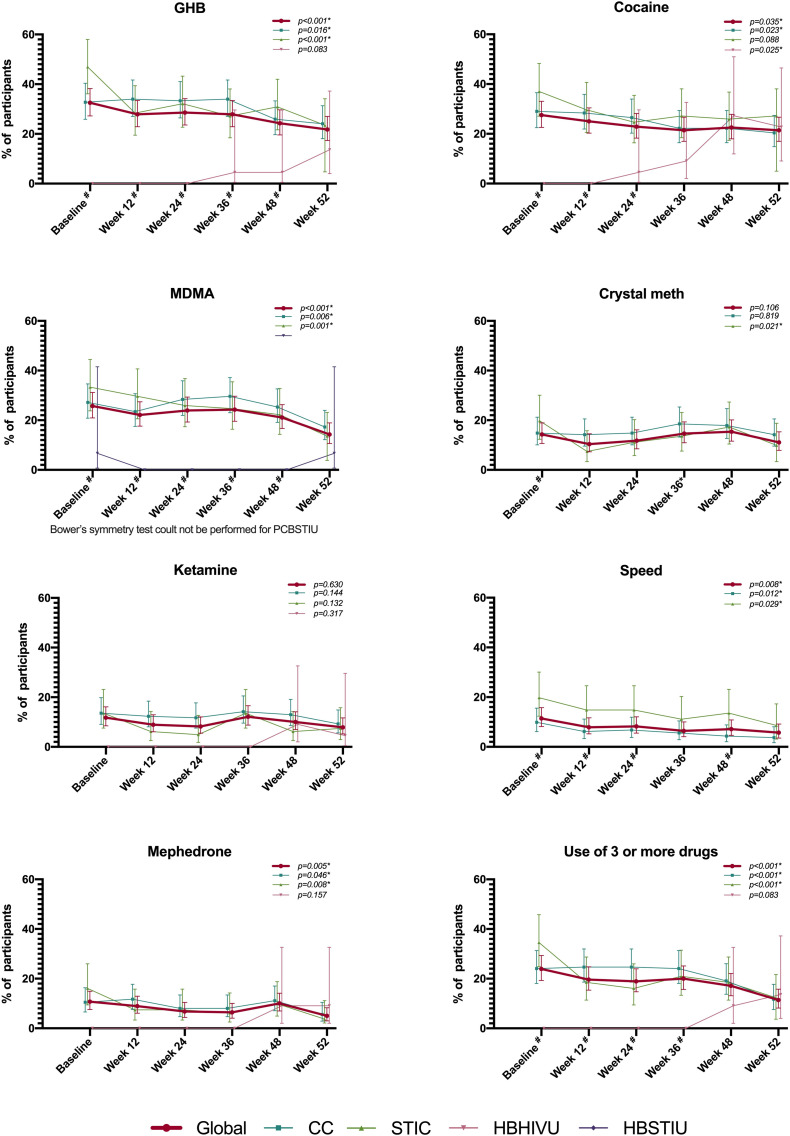
Frequency of drug use. # p<0.005 for Chi-squared test for percentage difference between centres. Centres with no cases along all the study period are not represented graphicaly, but they have been considered for chi-squared test. p-values showed on the graph areas refer to the symmetry Bowker test to test the signifance of the increase between baseline and week 52 measures. * refers to statistically significant p-values.

Overall, GHB was the most frequently used drug at baseline (32.5%; 95%CI: 27.2‒38.2). This percentage significantly decreased to 21.8% (95%CI: 17.3‒27.0) at week 52. This decreasing trend was also significant for CC and STIC participants. Cocaine was used by 27.5% of all participants at baseline (95%CI: 22.6‒33.1), ranging to 21.4 (95%CI: 17.0 ‒26.7) at week 52, with this decrease being significant both globally and for CC, while it rose from 0 to 22.8% (95%CI: 9.1‒46.5) at HBHIVU. MDMA was used by 25.7% of all participants at baseline (95%CI: 21.0‒31.2) and 14.3% (95%CI: 10.1‒19.0) at week 52. This decline was significant globally and for CC and STIC participants. In total, 14.3% (95%CI: 10.6‒18.9) of participants used crystal meth at baseline. The overall frequency of use did not change significantly at week 52, but it did for STIC participants, decreasing from 19.8% (95%CI: 12.3‒30.1) at baseline to 9.9% (95%CI: 3.3‒18.8) at week 52. Ketamine use remained stable during the study period as well (overall frequency of use at baseline 11.8%; 95%CI: 8.5‒16.2). Some 11.4% of all the participants used speed at baseline (95%CI: 8.2‒15.8), decreasing significantly to 5.7% at week 52 (95%CI: 3.5‒9.2), as happened among CC and STIC participants. Overall mephedrone use declined from 10.7% (95%CI: 7.6‒15.0) at baseline to 5.0% (95%CI: 3.0‒8.3) at week 52. This decrease was also observed for CC and STIC. Lastly, the overall frequency of use of three or more of these drugs was 23.9% (95%CI: 19.3‒29.3) at baseline, showing a significant decrease to 11.4% (95%CI: 8.2‒15.8) at week 52. This reduction was also found to be significant at CC and STIC (Fig 2).

### Participants’ and professionals’ satisfaction

Overall, 97.9% of participants indicated that they had the highest degree of agreement when asked if waiting times had been appropriate, with the highest percentage at the HBSTIU (100%) and the lowest at the HBHIVU (86.4%) (p<0.001). When participants were asked if they had received proper information about how to collect PrEP pills, 97.9% responded with the highest degree of agreement, and some 97.9% had the highest degree of satisfaction regarding the information they received about what to do in the case of presenting side effects, with no differences by centres. Lastly, general satisfaction with PrEP was awarded the highest degree of satisfaction by 98.6% of participants.

Similarly, the four study centres showed the highest degree of agreement with items 1 to 4. Only the CC had the highest degree of agreement with item 5 (“the centre has enough personnel to conduct the study”), while the STIC, the HBHIVU and the HBSTIU said they only moderately agreed with it.

## Discussion

After one year of follow-up, no seroconversion has occurred among participants and side effects have been rare or mild, showing that PrEP is an effective and safe preventive strategy, as has been shown before [2, 3].

Unfortunately, we did not achieve the intended sample size of 400 participants, with differences in the number of patients recruited between study centres. Although epidemiology could partially explain the higher numbers from the centres in Barcelona (CC and STIC), other factors may be involved. HIV units placed in hospitals may not have as easy access to potential PrEP candidates as community centres and STI centres, which are already familiar to potential PrEP users. In this regard, distrust of health-care providers has been identified as a barrier to wider PrEP use in the USA [13], where PrEP has been available since 2014. Furthermore, in a study conducted in 2017, we found that those living in smaller cities were less likely to be aware of PrEP [14].

Moreover, requiring at least two risk behaviours as inclusion criteria could keep some people still at risk of HIV infection from being recruited in the study. Unfortunately, we did not collect information about the number and reasons why potential participants were not recruited, other than HIV diagnosis during screening. We strongly recommend collecting this valuable information in real life PrEP cohorts in order to assess the number of people that are not engaging with PrEP because of strict inclusion requirements. Additionally, we recruited only three TW, which indicates that specific strategies are needed to engage this key population in PrEP, as has been already discussed in other contexts [15].

The overall retention rate was 87.2%, similar to other studies [PRELUDE: 81% [16]; PrEP Brazil study: 83% [17]]. The only factor related to PrEP abandonment (for any reason) that we found was the study site, with higher odds of abandonment among participants from the HBHIVU and the HBSTIU. This could also be related with mistrust of healthcare providers: CC and STIC count with personnel specialized in sexual health and trained on cultural competences. These sites may be perceived as more friendly resources than hospital-based ones. Collaborating with community organizations and STI centres has already been identified as a potential facilitator in gaining access to PrEP in Spain [12].

We measured adherence using the SMAQ scale, which classified 75.9% and 85.9% of participants as adherent at weeks 4 and 52, respectively. However, we found this tool was not ideal for adherence monitoring, since four of the items do no refer to any time frame and therefore we analysed the one item referring to a time frame (number of doses missed in the last week) independently. Using this item, overall adherence was optimal from week 4 and did not change at week 52. HBSTIU showed a lower frequency of participants not missing any dose at week 52, but none of them missed more than two pills, which would provide protective levels, following the results of iPrEx OLE [18]. Self-reported measures have proved to have good correlation with laboratory measures, especially those related to the last 4 weeks, rather than longer periods [19]. Based on our experience, we would recommend using specific self-reported measures to measure PrEP adherence instead of general tools such as SMAQ.

The overall STI rates both at baseline and at the end of the study were higher than those found in the general population [20] as we expected since having an STI was an inclusion criterion. Although the frequency of condom use decreased, we observed a significant increase only for syphilis among CC participants (0% to 4.9%) and for chlamydia among STIC participants (3.7% to 12.4%). A lack of association between condom use and STI increments was found in one Australian cohort, maybe due to other unknown or unmeasured risk behaviours such as an increase in having new sexual partners [21]. Nevertheless, the relation between having an STI and PrEP use has been controversial: while some studies have found that PrEP use would increase STI incidence [22–24], others show that PrEP would not lead to STI increases or would even lower STI rates, due to frequent testing and close follow-up of sexual health [25]. This would be consistent with the fact that having STIs and not using condoms consistently in the last 12 months were inclusion criteria for the study, as well as being and indicative criteria in the national guidelines. Additionally, some studies found that most new STI infections were diagnosed in non-study visits [26]. This should be taken into consideration by PrEP programmes during implementation and scaling up, in order not to underestimate STI incidence.

We found an STI incidence of 68.4 (95%CI:59.5–80.0), consistent with that found in other observational studies [17, 21, 26–28]. However, since one of the study sites did not perform throat and anal swabs systematically, this figure may be underestimated.

An important finding from our study is the decrease in drug use after one year on PrEP, which was significant in at least one study centre for GHB, cocaine, MDMA, crystal meth, mephedrone and for the use of three or more drugs. However, participants from the HBHIVU showed the opposite trend, although it was only significant for cocaine, which rose from 0% at baseline to 22% at week 52. We believe that this finding could be jeopardized by a lack of drug use disclosure among HBHIVU participants, since they were not familiar with the health-care professionals from the study, unlike the rest of the participants. A reduction in drug use could be related to lower anxiety related to possible HIV infection, as has already been described for PrEP users in Australia [29]. These findings underline the capacity of PrEP programmes to approach sexual health comprehensively. Moreover, although we did not collect this information, some of these drugs are frequently used in chemsex [30], making it possible that a reduction in the practice of chemsex also occurred among participants. In order to be able to monitor changes in this regard, we would recommend that PrEP programmes collect information about chemsex.

In regards to satisfaction, although patients’ perception was generally good, it is necessary to further explore the needs of potential PrEP sites, such as the lack of personnel demonstrated in our results. As we understand it, setting up PrEP programmes entails an increase in the number of users and procedures at health-care settings that would need an increase in those health-care settings’ resources.

Lastly, the performance of this study enhanced the decision-making process and facilitated PrEP implementation in Spain. In fact, the MOH considered the preliminary results in making recommendations that have been published in the national PrEP implementation guidelines [31].

This study has some limitations. The size of the sample was small, especially in HBHIU and HBSTIU, providing some imprecise estimations for some outcomes. However, this showed us that accessing people in need of PrEP may be more difficult in some settings where extra effort must be made to reach this population. Many participants were LTFU and could not be reached to discover the real reasons why they discontinued PrEP. We acknowledge that satisfaction measures did not explore the barriers that could be involved in PrEP implementation and scaling up in Spain in depth; further research needs to be conducted to address this need for evidence. Despite these limitations, we believe that our study provides useful evidence to be considered in PrEP implementation and scaling up in Spain and similar contexts.

In conclusion, our results showed that PrEP implementation is feasible in four different types of health-care settings within the NHS. CC, STIC, HBHIVU and HBSTIU have established the necessary circuits to conduct the study and obtained good outcomes on retention, safety, efficacy and satisfaction. Health policymakers must consider the epidemiological and structural specificities of their context to make sure that PrEP is accessible, and identify whether the health-care centres that will start PrEP programmes have sufficient resources. PrEP programmes should include information systems capable of properly collecting and monitoring accessibility, retention, reasons for discontinuations, adherence and sexual behaviours. Our findings about the lack of a consistent increase of STIs and the decrease in drug use underline the fact that PrEP programmes may be able to approach sexual health more broadly than just through HIV prevention.

## Supporting information

S1 Appendix Study questionnaires. (PDF)

S1 Fig Study flow chart. (PDF)
