# Supplementary material for: Implementation of pre-exposure prophylaxis programme in Spain. Feasibility of four different delivery models
Source: PLoS One. 2021 Feb 8;16(2):e0246129. doi: 10.1371/journal.pone.0246129 (PMC7870089; doi:10.1371/journal.pone.0246129)
Supplement: S1 Appendix — (PDF) [file pone.0246129.s001.pdf]

S1 Appendix. Study questionnaires

Behavioural variables

**Variable: Condom use during sexual intercourse (Spanish):**
¿Con qué frecuencia usa el preservativo durante sus relaciones sexuales?
Nunca
Ocasionalmente
Habitualmente
Siempre

**Variable: Condom use during sexual intercourse (English):**
How often do you use condom during sexual intercourse?
Never
Sometimes
Often
Always

**Variable: Regular use of drugs (Spanish):**
¿Cuáles de las siguientes drogas utilizas de manera habitual?
Alcohol
Cannabis
GHB/GBL
Poppers
MDMA
Ketamina
Mefedrona
Speed
Metanfetamina
Otras drogas

**Variable: Regular use of drugs (English):**
Which of the following do you take regularly?
Alcohol
Cannabis
GHB/GBL
Poppers
MDMA
Ketamine
Mephedrone
Speed
Metanphetamine
Other drugs

PrEP related measures

**Variable: Number of missed doses (Spanish)**
En la última semana, ¿cuántas veces has olvidado tomar la PrEP?
Ninguna
1-2
3-4
5-7

**Variable: Number of missed doses (English)**
How many PrEP did you miss in the last week?
None
1-2
3-4
5-7

Participant satisfaction

**Variable: Satisfaction with waiting times (Spanish)**
¿El tiempo de espera ha sido adecuado?
Completamente en desacuerdo
En desacuerdo
De acuerdo
Completamente de acuerdo

**Variable: Satisfaction with waiting times (English)**
Was waiting time appropiate?
Strongly disagree
Disagree
Agree
Strongly agree

**Variable: Satisfaction with the information received about collecting PrEP pills (Spanish)**
¿La información recibida sobre cómo recoger la PrEP ha sido adecuada?
Completamente en desacuerdo
En desacuerdo
De acuerdo
Completamente de acuerdo

**Variable: Satisfaction with the information received about collecting PrEP pills (English)**
Was the information received about collecting PrEP pills appropiate?
Strongly disagree
Disagree
Agree
Strongly agree

**Variable: Satisfaction with the information received about what to do in the case of side effects (Spanish)**
¿La información recibida sobre cómo actuar en casa de efectos adversos ha sido adecuada?
Completamente en desacuerdo
En desacuerdo
De acuerdo
Completamente de acuerdo

**Variable: Satisfaction with the information received about what to do in the case of side effects (English)**
Was the information received about what to do in the case of side effects appropiate?
Strongly disagree

Disagree
Agree
Strongly agree

**Variable: General satisfaction with PrEP (Spanish)**
De manera general ¿qué grado de satisfacción tienes con respecto a la PrEP en este estudio?
Muy satisfecho
Satisfecho
Neutral
Insatisfecho

**Variable: General satisfaction with PrEP (English)**
How would you rate your experience with PrEP in general in this study?
Very satisfied
Satisfied
Neutral
Dissatisfied

Practitioner satisfaction

**Variable: Practitioner satisfaction (Spanish)**				
Por favor, indica tu nivel de acuerdo con cada uno de los siguientes enunciados				
La aceptación de los participantes a participar en el estudio ha sido la esperada	Completamente de acuerdo	De acuerdo	En desacuerdo	Completamente de acuerdo
La proporción de participantes que cumplió criterios de PrEP fue la esperada	Completamente de acuerdo	De acuerdo	En desacuerdo	Completamente de acuerdo
Las instalaciones del centro permitieron mantener la privacidad durante las visitas del estudio	Completamente de acuerdo	De acuerdo	En desacuerdo	Completamente de acuerdo
La relación con el hospital ha sido adecuada	Completamente de acuerdo	De acuerdo	En desacuerdo	Completamente de acuerdo
El centro ha contado con personal suficiente necesario para llevar a cabo el estudio	Completamente de acuerdo	De acuerdo	En desacuerdo	Completamente de acuerdo

**Variable: Practitioner satisfaction (English)**				
Pleases, assess your degree of agreement with the folowing statements:				
Participants' willingness to participate in the study was as high as expected	Strongly agree	Agree	Disagree	Strongly disagree
The proportion of participants meeting	Strongly agree	Agree	Disagree	Strongly disagree

PrEP criteria was similar to that expected				
The centre's facilities allowed privacy to be maintained during visits	Strongly agree	Agree	Disagree	Strongly disagree
The relationship with the hospital has been appropriate	Strongly agree	Agree	Disagree	Strongly disagree
The centre has enough personnel to conduct the study	Strongly agree	Agree	Disagree	Strongly disagree
