# Supplementary figures and images for: Implementation of pre-exposure prophylaxis programme in Spain. Feasibility of four different delivery models
Source: PLoS One. 2021 Feb 8;16(2):e0246129. doi: 10.1371/journal.pone.0246129 (PMC7870089; doi:10.1371/journal.pone.0246129)

**S1 Figure. Study flow chart**

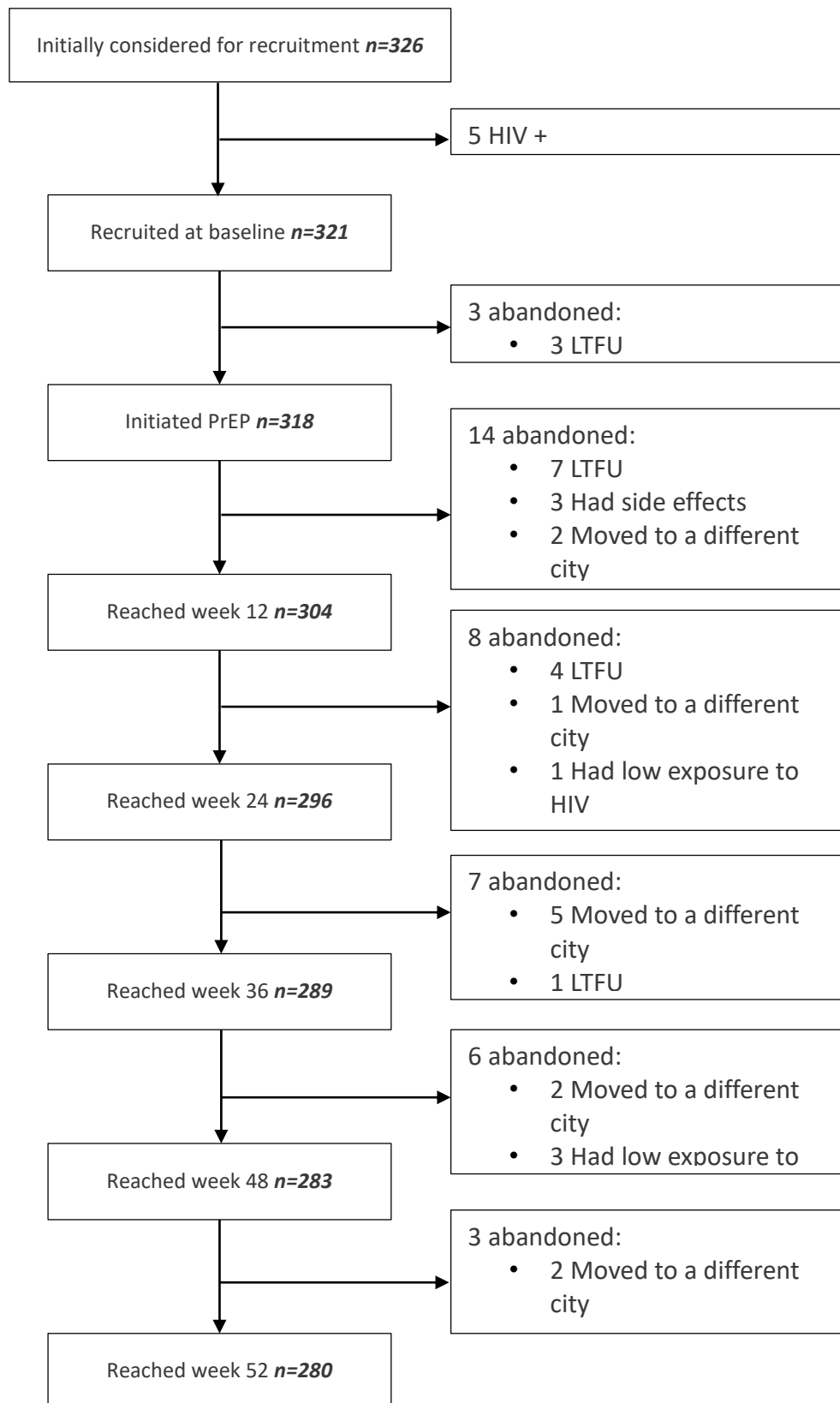

Supplement: S1 Fig — (PDF) [file pone.0246129.s002.pdf]
